# Supplementary material for: Inactivation of the Hippo tumor suppressor pathway promotes melanoma
Source: Nat Commun. 2022 Jun 29;13:3732. doi: 10.1038/s41467-022-31399-w (PMC9243107; doi:10.1038/s41467-022-31399-w)
Supplement: Supplementary file 1 — Supplementary Information [file 41467_2022_31399_MOESM1_ESM.pdf]

Inactivation of the Hippo Tumor Suppressor Pathway Promotes Melanoma

Marc A. Vittoria[1], Nathan Kingston[2], Kristyna Kotynkova[1], Eric Xia[1], Rui Hong[4], Lee Huang[3], Shayna McDonald[1], Andrew Tilston-Lunel[2], Revati Darp[5], Joshua Campbell[4], Deborah Lang[3], Xiaowei Xu[6], Craig Ceol[5], Xaralabos Varelas[2] and Neil J. Ganem[1,4]

[1]Department of Pharmacology & Experimental Therapeutics, Boston University School of Medicine, Boston, MA 02118, USA

[2]Department of Biochemistry, Boston University School of Medicine, Boston, MA 02118, USA

[3]Department of Dermatology, Boston University School of Medicine, Boston, MA 02118, USA

[4]Department of Medicine, Boston University School of Medicine, Boston, MA 02118, USA.

[5]Program in Molecular Medicine, University of Massachusetts Medical School, Worcester, MA 01605, USA

[6]Department of Pathology and Laboratory Medicine, University of Pennsylvania Perelman School of Medicine, Philadelphia, PA 19104, USA

Supplementary Information

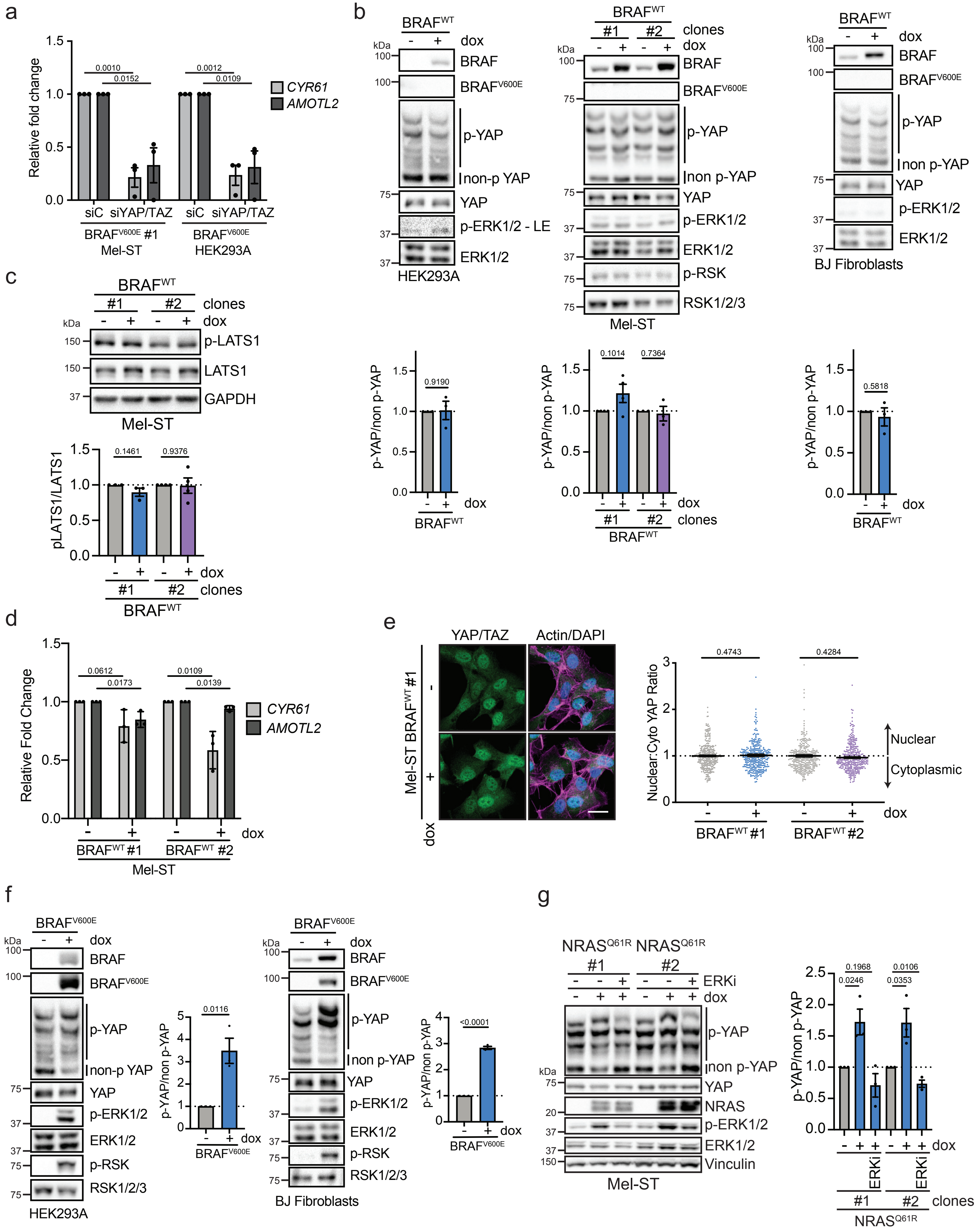
a
Relative fold change
1.5
1.0
0.5
0.0
0.0010
0.0152
0.0012
0.0109
CYR61
AMOTL2
siC
siYAP/TAZ
BRAFV600E #1
Mel-ST
BRAFV600E
HEK293A
b
BRAFWT
dox
kDa
BRAF
BRAFV600E
p-YAP
non-p YAP
YAP
p-ERK1/2 - LE
ERK1/2
HEK293A
clones
#1
#2
non p-YAP
p-ERK1/2
p-RSK
RSK1/2/3
Mel-ST
BJ Fibroblasts
p-YAP/non p-YAP
0.9190
0.1014
0.7364
0.5818
c
p-LATS1
LATS1
GAPDH
pLATS1/LATS1
0.1461
0.9376
d
Relative Fold Change
0.0612
0.0173
0.0109
0.0139
BRAFWT #1
BRAFWT #2
e
YAP/TAZ
Actin/DAPI
Mel-ST BRAFWT#1
Nuclear:Cyto YAP Ratio
0.4743
0.4284
Nuclear
Cytoplasmic
f
0.0116
<0.0001
g
NRASQ61R
ERKi
NRAS
Vinculin
0.1968
0.0246
0.0106
0.0353

Supplemental Figure 1: Hyperactive MAPK Signaling Activates the Hippo Pathway (a) Relative expression of indicated genes from RT-PCR in dox-inducible *BRAF*^{V600E} Mel-ST and HEK293A cells treated with control siRNA (siC) or YAP and TAZ siRNAs (siYAP/TAZ) without dox (n = 3 independent experiments, graph shows mean ± SEM, two-tailed unpaired t test). **(b)** Representative IB of dox-inducible wild-type *BRAF* (*BRAF*^{WT}) in HEK293A, Mel-ST, and BJ fibroblast cell lines with intensity quantification of YAP phos-tag below (n = 3 independent experiments, graphs show mean relative intensity ± SEM, two-tailed unpaired t test). **(c)** IB of dox-inducible *BRAF*^{WT} Mel-ST clones cultured ± dox for 24 h (n ≥ 3 independent experiments, graph shows mean relative intensity ± SEM, two-tailed unpaired t test). **(d)** Relative expression of indicated genes from RT-PCR in *BRAF*^{WT} Mel-ST clones cultured ± dox for 24 h (n = 3 independent experiments, graph shows mean ± SEM, two-tailed unpaired t test). **(e)** Left, representative immunofluorescence image of *BRAF*^{WT} Mel-ST clone stained for YAP/TAZ (green) alone or merged with DNA (DAPI, blue) and actin (Phalloidin, magenta); Right, quantification of nuclear to cytoplasmic ratio of mean YAP/TAZ fluorescence (n > 300 cells from three independent experiments, graph shows mean ± SEM, scale bar = 25 μm, two-tailed Mann-Whitney test). **(f)** Representative IB of dox-inducible *BRAF*^{V600E} HEK293A and BJ cell lines with intensity quantification of YAP phos-tags (n = 3 independent experiments, graphs show mean ± SEM, two-tailed unpaired t test). **(g)** Left, representative IB of dox-inducible *NRAS*^{Q61R} Mel-ST clones cultured ± dox for 24 h; Right, intensity quantification of YAP phos-tag (n = 3 independent experiments, graph shows mean ± SEM, two-tailed unpaired t test). Source Data are provided as a Source Data file.

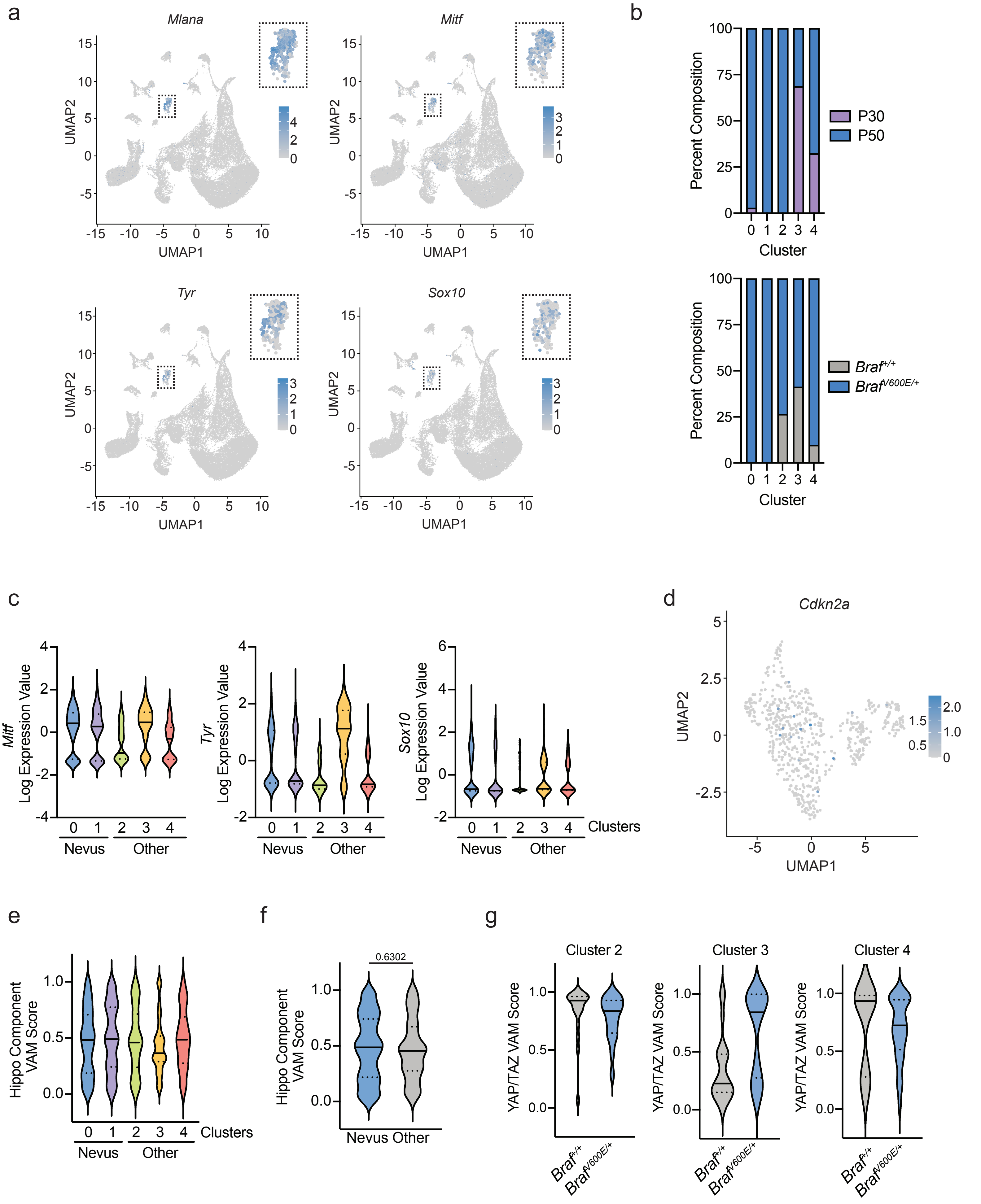

a
Mlana
Mitf
Tyr
Sox10
UMAP2
UMAP1
b
Percent Composition
Cluster
P30
P50
Braf+/+
BrafV600E/+
c
Mitf Log Expression Value
Tyr Log Expression Value
Sox10 Log Expression Value
Nevus
Other
Clusters
d
Cdkn2a
UMAP2
UMAP1
e
Hippo Component VAM Score
Nevus
Other
Clusters
f
0.6302
Hippo Component VAM Score
Nevus Other
g
Cluster 2
Cluster 3
Cluster 4
YAP/TAZ VAM Score
Braf+/+
BrafV600E/+

Supplemental Figure 2: Detailed Analysis of GSE154679 (a) UMAP of all single-cells from nevus containing murine skin from GSE154679 displaying relative expression of melanocyte markers *Mlana*, *Mitf*, *Tyr*, *Sox10*. **(b)** Bar graphs of melanocyte subcluster composition dependent upon animal age (top) or genotype (bottom). **(c)** Log expression of indicated genes across melanocyte subclusters. **(d)** UMAP of *Cdkn2a* log expression across melanocyte subclusters (n = 589). **(e)** Hippo component VAM score plotted by melanocyte subcluster (n = 589). **(f)** Hippo component VAM score comparing nevus (clusters 0, 1) and other melanocytes (clusters 2, 3, 4) (nevus n = 408, other n = 181, two-tailed Mann-Whitney test). **(g)** YAP/TAZ VAM score according to indicated melanocytic subcluster and genotype. Source Data are provided as a Source Data file.

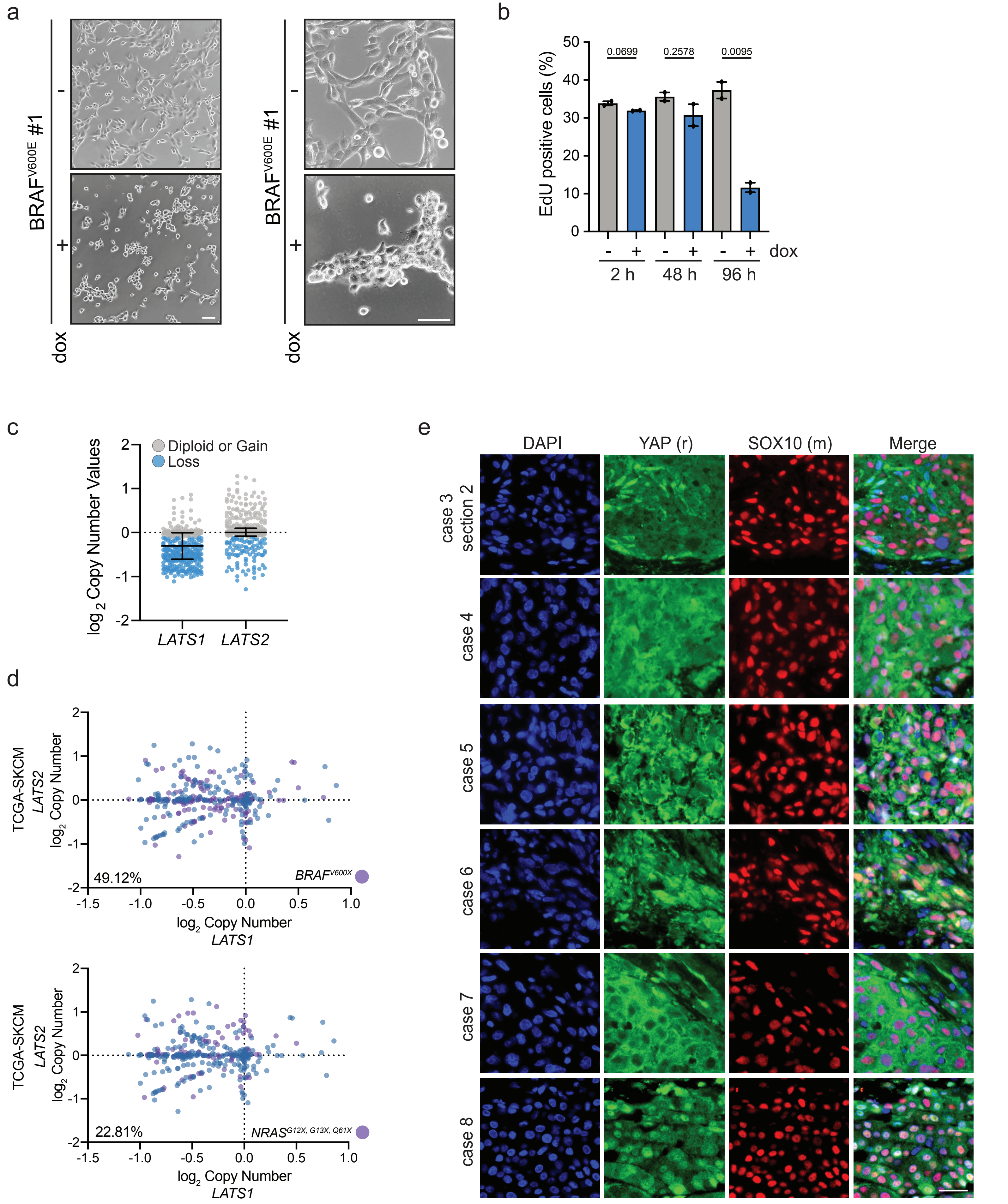

a
BRAF^{V600E} #1
-
+
dox
b
EdU positive cells (%)
0.0699
0.2578
0.0095
dox
2 h
48 h
96 h
c
log$_2$ Copy Number Values
Diploid or Gain
Loss
LATS1
LATS2
d
TCGA-SKCM
LATS2
log$_2$ Copy Number
49.12%
BRAF^{V600X}
log$_2$ Copy Number
LATS1
22.81%
NRAS$^{G12X, G13X, Q61X}$
e
DAPI
YAP (r)
SOX10 (m)
Merge
case 3 section 2
case 4
case 5
case 6
case 7
case 8

Supplemental Figure 3: A Subset of Human Melanoma Demonstrates Hippo Pathway Inactivation (a) Representative phase-contrast images of a dox-inducible *BRAF*^{V600E} Mel-ST clone ± dox for 48 h at 4X magnification; Right, 10X magnification (scale bar = 100 μm). **(b)** Percent EdU positive dox-inducible *BRAF*^{V600E} Mel-ST cells ± dox for indicated time (n > 200 cells per condition from 2 independent experiments, graph shows mean ± SEM, two-tailed unpaired t test). **(c)** Plot of log_2 copy number values from TCGA-SKCM for indicated genes (n = 367, bars represent median with interquartile range). **(d)** Same data as Figure 3F from TCGA-SKCM with purple circles representing tumors with indicated mutations; bottom left percent is frequency of *LATS1/2* co-heterozygous loss that occurs with indicated mutations. **(e)** Representative immunofluorescence staining of indicated proteins in panel of human melanoma tissue (scale bar = 25 μm). Source Data are provided as a Source Data file.

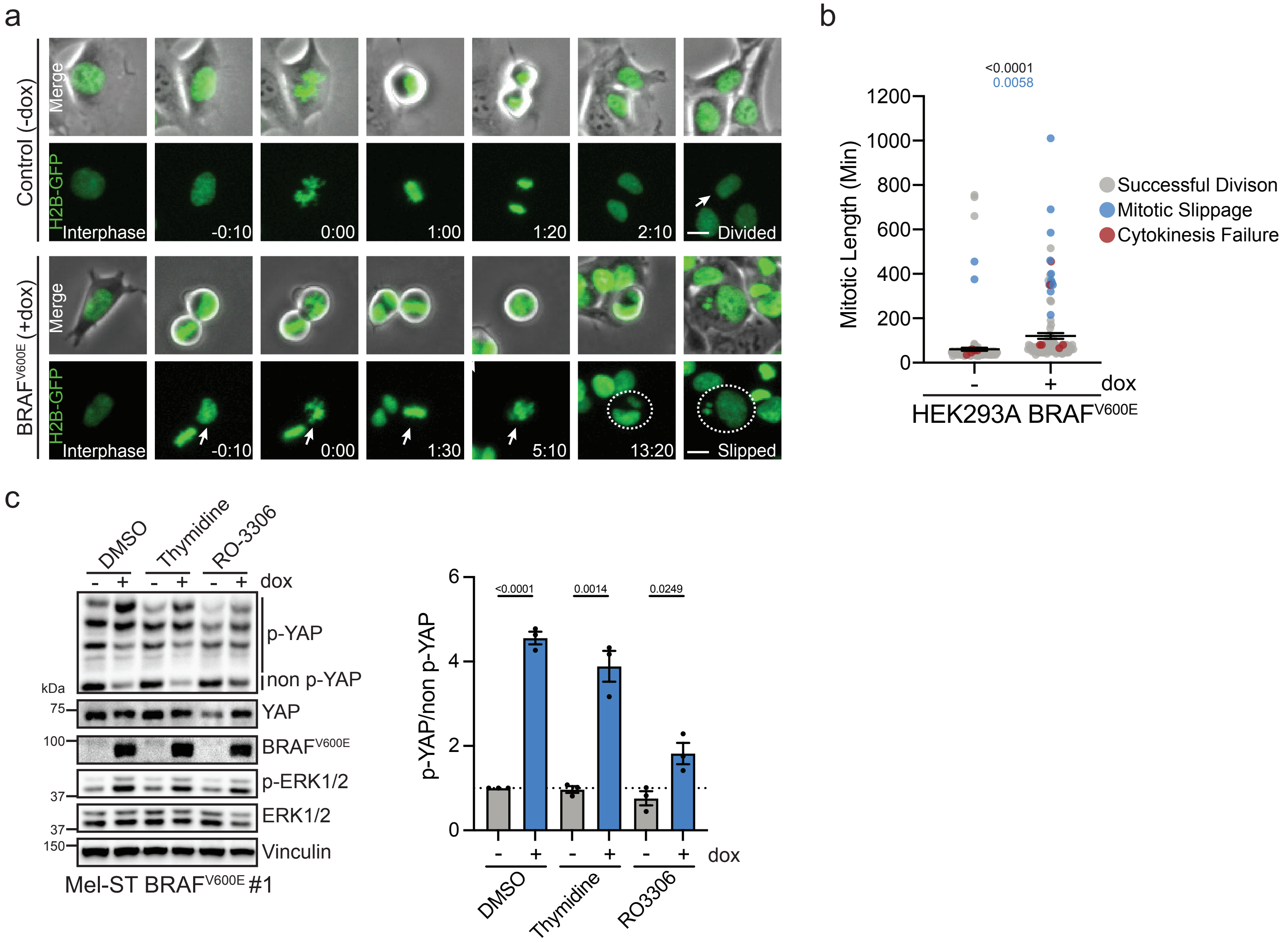

a
Control (-dox)
Merge
H2B-GFP
Interphase
-0:10
0:00
1:00
1:20
2:10
Divided
BRAFV600E (+dox)
Merge
H2B-GFP
Interphase
-0:10
0:00
1:30
5:10
13:20
Slipped
b
<0.0001
0.0058
Mitotic Length (Min)
1200
1000
800
600
400
200
0
Successful Divison
Mitotic Slippage
Cytokinesis Failure
-
+
dox
HEK293A BRAFV600E
c
DMSO
Thymidine
RO-3306
- + - + - +
dox
p-YAP
non p-YAP
kDa
75
100
37
37
150
YAP
BRAFV600E
p-ERK1/2
ERK1/2
Vinculin
Mel-ST BRAFV600E #1
p-YAP/non p-YAP
6
4
2
0
<0.0001
0.0014
0.0249
- + - + - +
dox
DMSO
Thymidine
RO3306

Supplemental Figure 4: Oncogenic BRAF Promotes Mitotic Dysfunction (a) Representative still fluorescence and phase-contrast images from a live-cell video of H2B-GFP (green) expressing dox-inducible $BRAF^{V600E}$ HEK293A cells cultured ± dox (scale bar = 25 μm, hh:mm). **(b)** Plot of mitotic duration and fate of individually tracked mitoses from (a) (n > 120 mitoses per condition from two independent experiments, dots represent individually tracked mitoses, black p-value represents mitotic length significance, two-tailed unpaired t test, blue p-value represents significance for difference in frequency of whole-genome doubling events, two-sided Fisher's exact test). **(c)** IB of dox-inducible $BRAF^{V600E}$ Mel-ST cells treated with Thymidine 2.5 mM or RO-3306 7 μM; Right, intensity quantification of YAP phos-tag (n = 3 independent experiments, graph shows mean ± SEM, two-tailed unpaired t test). Source Data are provided as a Source Data file.

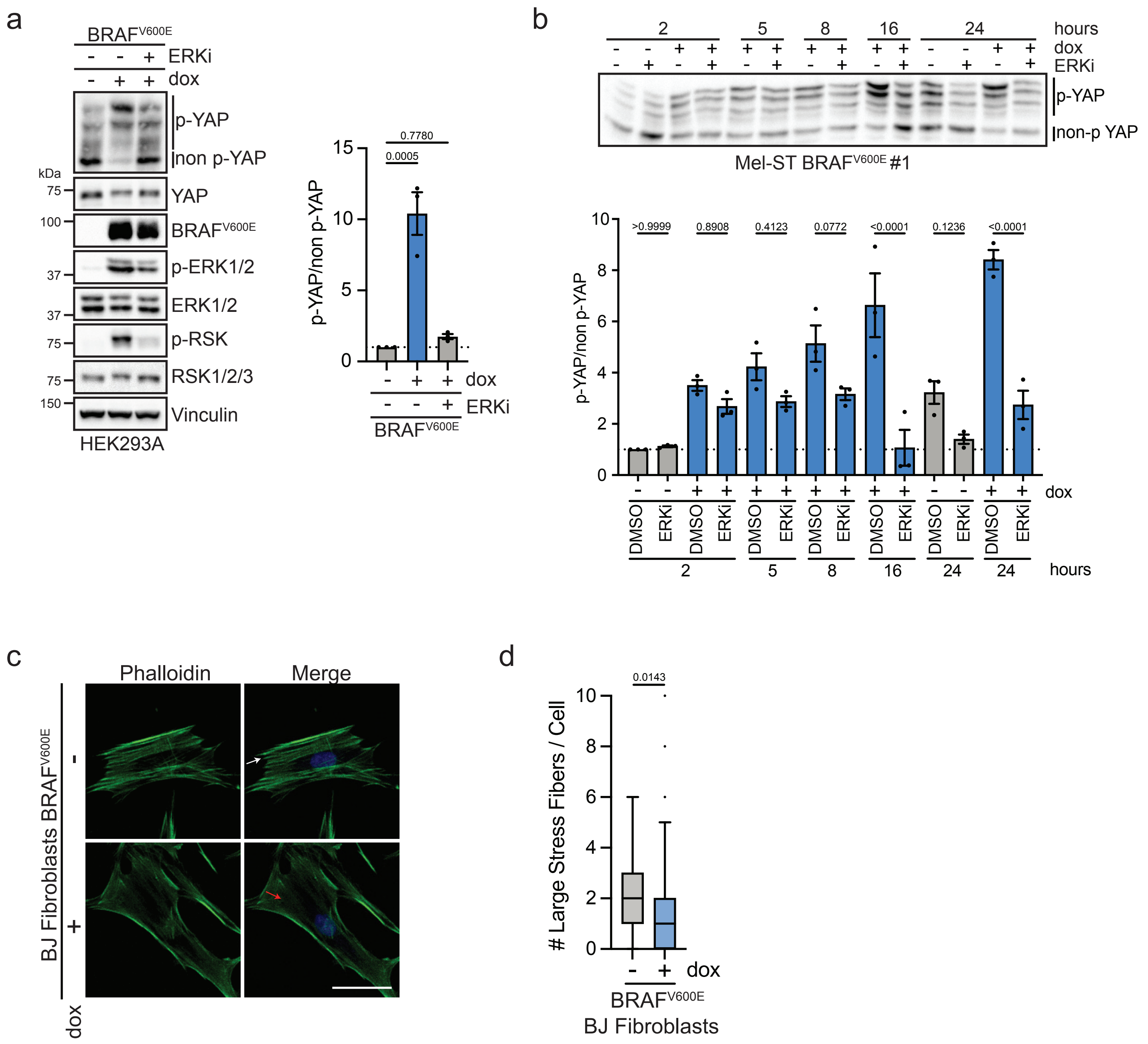
a
BRAF^V600E
- - + ERKi
- + + dox
p-YAP
non p-YAP
kDa
75
YAP
100
BRAF^V600E
37
p-ERK1/2
37
ERK1/2
75
p-RSK
75
RSK1/2/3
150
Vinculin
HEK293A
p-YAP/non p-YAP
15
10
5
0
0.7780
0.0005
- + + dox
- - + ERKi
BRAF^V600E
b
2 5 8 16 24 hours
- - + + + + + + + + - - + + dox
- + - + - + - + - + - + - + ERKi
p-YAP
non-p YAP
Mel-ST BRAF^V600E #1
p-YAP/non p-YAP
10
8
6
4
2
0
>0.9999
0.8908
0.4123
0.0772
<0.0001
0.1236
<0.0001
- - + + + + + + + + - - + + dox
DMSO ERKi DMSO ERKi DMSO ERKi DMSO ERKi DMSO ERKi DMSO ERKi DMSO ERKi
2 5 8 16 24 24 hours
c
Phalloidin
Merge
BJ Fibroblasts BRAF^V600E
-
+
dox
d
0.0143
Large Stress Fibers / Cell
10
8
6
4
2
0
- + dox
BRAF^V600E
BJ Fibroblasts

Supplemental Figure 5: Oncogenic BRAF Promotes Hippo Pathway Activation (a) IB of dox-inducible *BRAF*^{V600E} HEK293A cells treated with indicated drugs for 24 h; Right, intensity quantification of YAP phos-tag (n = 3 independent experiments, graph shows mean ± SEM, one-way ANOVA with Dunnett's multiple comparisons test). **(b)** IB of indicated dox-inducible *BRAF*^{V600E} Mel-ST clone treated with or without dox for 24 h then subsequently treated with indicated drugs for time course of 2 to 24 h; Below, intensity quantification of YAP phos-tag (n = 3 independent experiments, graph shows mean ± SEM, one-way ANOVA with Dunnett's multiple comparisons test). **(c)** Representative maximum intensity projections of confocal z-stacks of dox-inducible *BRAF*^{V600E} BJ Fibroblasts stained for phalloidin and DAPI. Top, white arrow denotes stress fiber; Bottom, red arrow denotes lack of stress fibers (n = 2 independent experiments, scale bar = 25 μm). **(d)** Quantification of large stress fibers per cell in Figure S5C (n > 140 cells per condition from 2 independent experiments, box is 25th to 75th percentile with line representing median, whiskers plotted via Tukey method, no dox: minima 0, 25th 1, median 2, 75th 4, maxima 6; dox: minima 0, 25th 0, median 1, 75th 2.0, maxima 10). Source Data are provided as a Source Data file.

a

Publication	Strain	4-HT Induction Technique	Time to Melanoma Formation	developed / total n	Time to Nevus Formation	developed / total n
Dankort et al, 2009, *Nature Genetics*	Tyr::CreER^{T2}, Braf$^{CA/+}$, Pten$^{+/+}$	Topical	Aged to > 800 days without tumor formation	0/22	~21-28 days	22/22
	Tyr::CreER^{T2}, Braf$^{+/+}$, Pten$^{lox4-5/lox4-5}$	Topical	Aged ~18 months without tumor formation	0/20	None observed	0/20
	Tyr::CreER^{T2}, Braf$^{CA/+}$, Pten$^{lox4-5/+}$	Topical	Not reported; Mice required euthanasia between 100 - 200 days	5/5	Not reported	5/5
	Tyr::CreER^{T2}, Braf$^{CA/+}$, Pten$^{lox4-5/lox4-5}$	Topical	Not reported; Mice required euthanasia between 25-50 days	22/22	Pigmented lesions rapidly became malignant	---
Ruiz-Vega et al, 2020, *eLife*	Tyr::CreER^{T2}, Braf$^{CA/+}$	Topical	Length of aging not reported, no tumor formation noted	none, n not reported	~14-21 days	all mice, n not reported

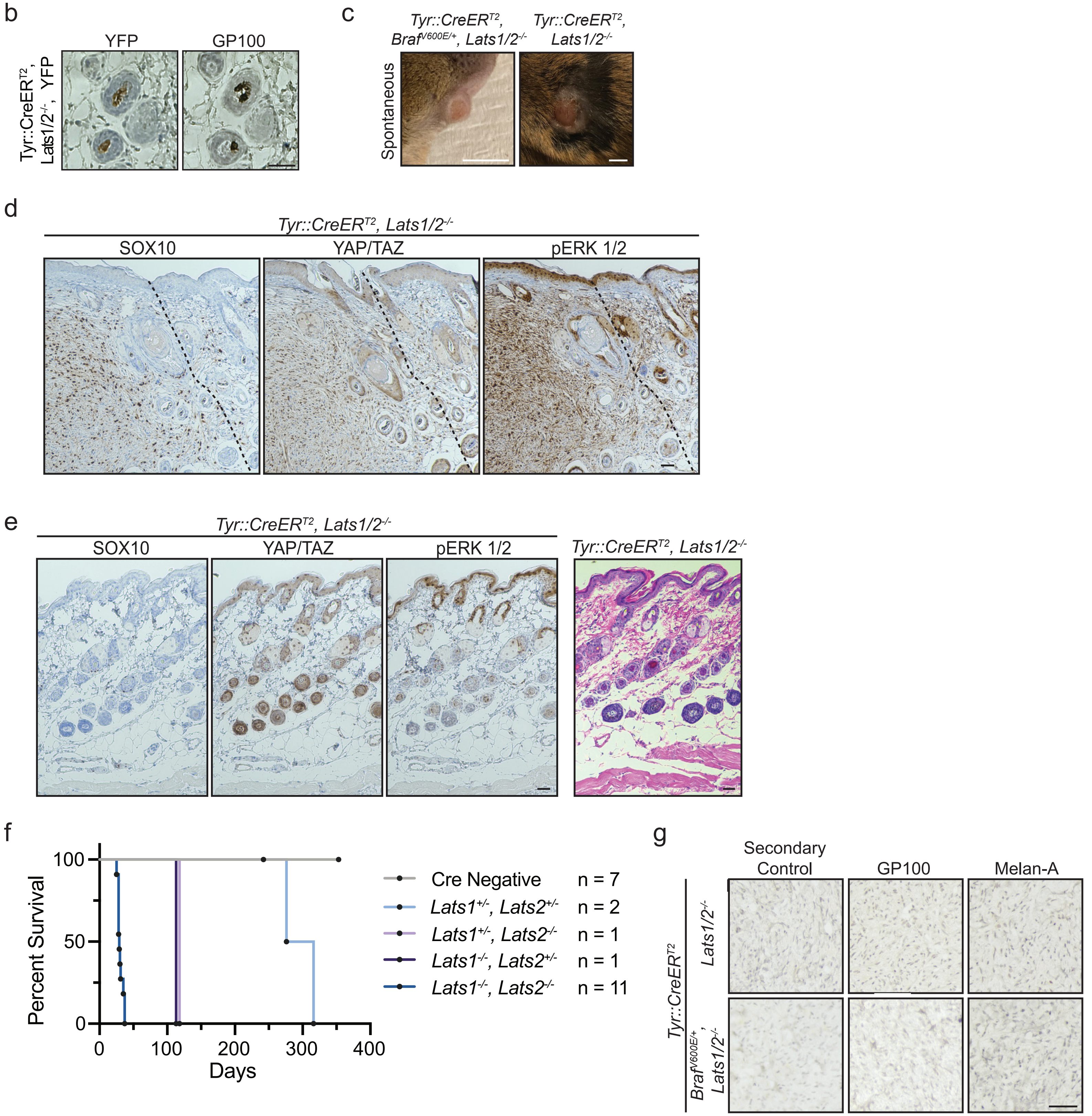

Supplemental Figure 6: Characterization of *Lats1/2*$^{-/-}$ Melanoma (a) Table of publications with rate of nevus or melanoma formation in various *Braf*^{CA}-expressing mice. **(b)** Representative IHC of indicated proteins, scale bar = 40 µm (n ≥ 2 independent mice with similar results). **(c)** Representative images of spontaneous *Braf*^{V600E}*/Lats1/2*$^{-/-}$ or *Lats1/2*$^{-/-}$ mouse tumors, scale bars = 0.5 cm. **(d)** Representative IHC of indicated proteins in *Lats1/2*$^{-/-}$ tumor (left of dotted line) and normal adjacent tissues (right of dotted line), scale bar = 40 µm, n ≥ 3 mice with similar results. **(e)** Representative IHC of indicated proteins of *Lats1/2*$^{-/-}$ mouse skin treated with 4-HT prior to tumorigenesis; right is a hematoxylin and eosin staining of a serial section, scale bar = 40 µm, n = 1 mouse. **(f)** Time from IP injection of tamoxifen to a study endpoint of mice with indicated genotypes. **(g)** Representative IHC of indicated proteins including secondary antibody only control, scale bar = 40 µm, n ≥ 2 mice with similar results. Source Data are provided as a Source Data file.

a

NAME	SIZE	ES	NES	NOM p-val	FDR q-val
CORDENONSI_YAP_CONSERVED_SIGNATURE	57	0.642258	2.051217	0	0
WANGETAL_YAPTAZ_TARGET_SCORE	22	0.764397	2.0402122	0	0
YAP1_UP	45	0.45573	1.4316236	0.054347824	0.11726231
REACTOME_YAP1_AND_WWTR1_TAZ_STIMULATED_GENE_EXPRESSION	15	0.336619	0.84074897	0.68662953	0.86892515
REACTOME_RUNX3_REGULATES_YAP1_MEDIATED_TRANSCRIPTION	8	0.347339	0.7453292	0.7538226	0.8074144

b

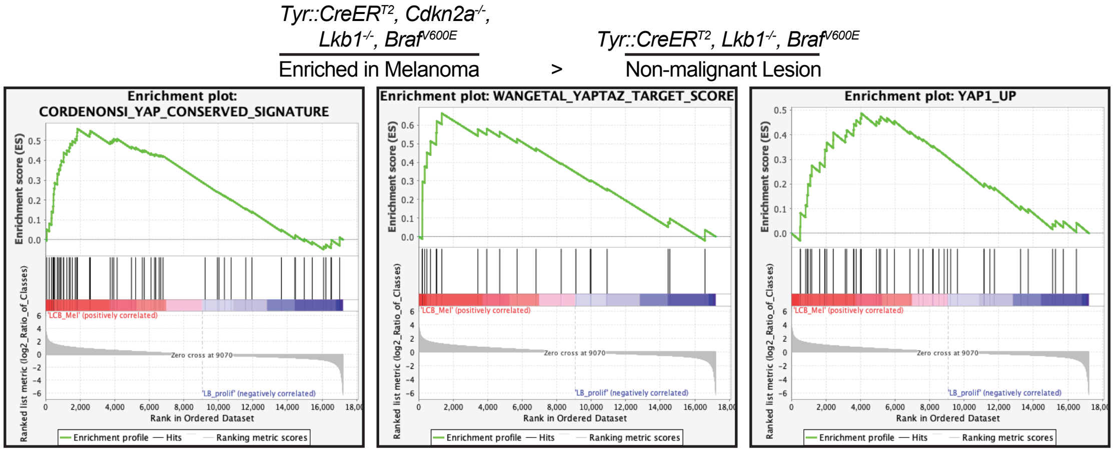

c

NAME	SIZE	ES	NES	NOM p-val	FDR q-val
CORDENONSI_YAP_CONSERVED_SIGNATURE	57	0.55971074	1.9228522	0	0
WANGETAL_YAPTAZ_TARGET_SCORE	22	0.66323537	1.866832	0.001477105	5.03E-04
YAP1_UP	45	0.48710054	1.5982525	0.005633803	0.01708542
REACTOME_RUNX3_REGULATES_YAP1_MEDIATED_TRANSCRIPTION	8	0.5892833	1.3134943	0.14194578	0.16061561
REACTOME_YAP1_AND_WWTR1_TAZ_STIMULATED_GENE_EXPRESSION	15	0.22996822	0.598145	0.95348835	0.9587438

d

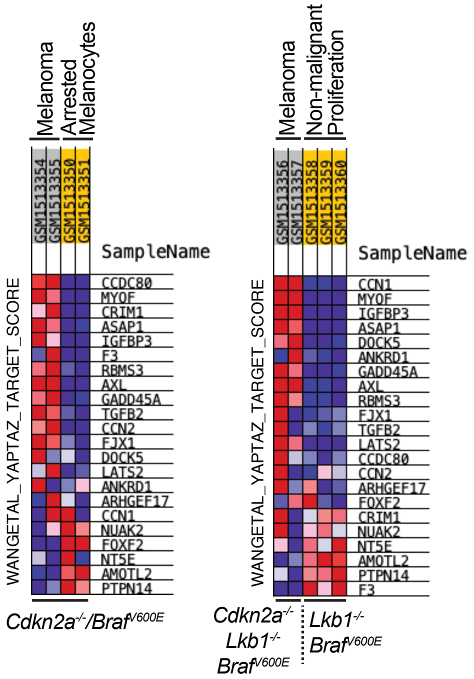

e

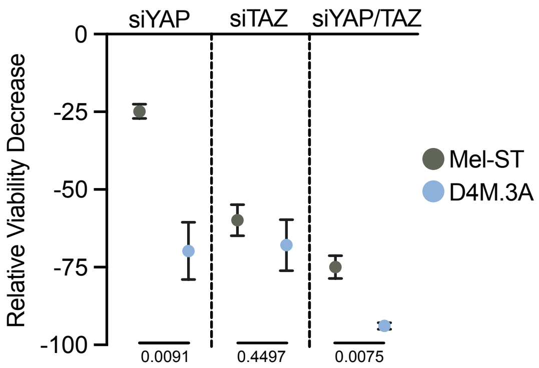

f

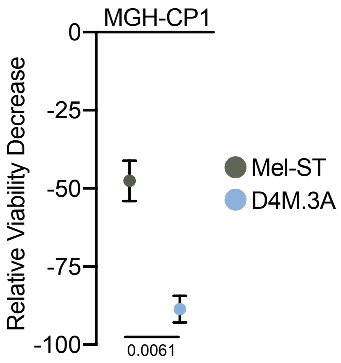

Supplemental Figure 7: YAP/TAZ Activation is Enriched in Animal Melanoma Models (a) Table of GSEA results from Figure 6A, see methods for full statistical analysis. **(b)** GSEA performed on GSE61750 comparing enrichment of YAP/TAZ signatures in melanoma to benign, proliferating melanocytes in indicated genotypes (see Figure S7D). **(c)** Table of GSEA results from Figure S7B. **(d)** GSEA heatmap results of gene expression from Figure 6A and S7B (red indicates higher expression, blue lower). **(e)** Percent relative viability decrease in indicated cell lines treated with siRNA targeting YAP (siYAP), TAZ (siTAZ) or a combination (siYAP/TAZ) for 4 days compared to control siRNA treated cells (n = 3 independent experiments with ≥ 5 technical replicates, graph shows mean ± SEM, two-tailed unpaired t test). **(f)** Relative viability of D4M.3A or Mel-ST cells treated with 10 μM TEAD inhibitor MGH-CP1 (n = 3 independent experiments with ≥ 5 technical replicates, graph shows mean ± SEM, two-tailed unpaired t test). Source Data are provided as a Source Data file.

a

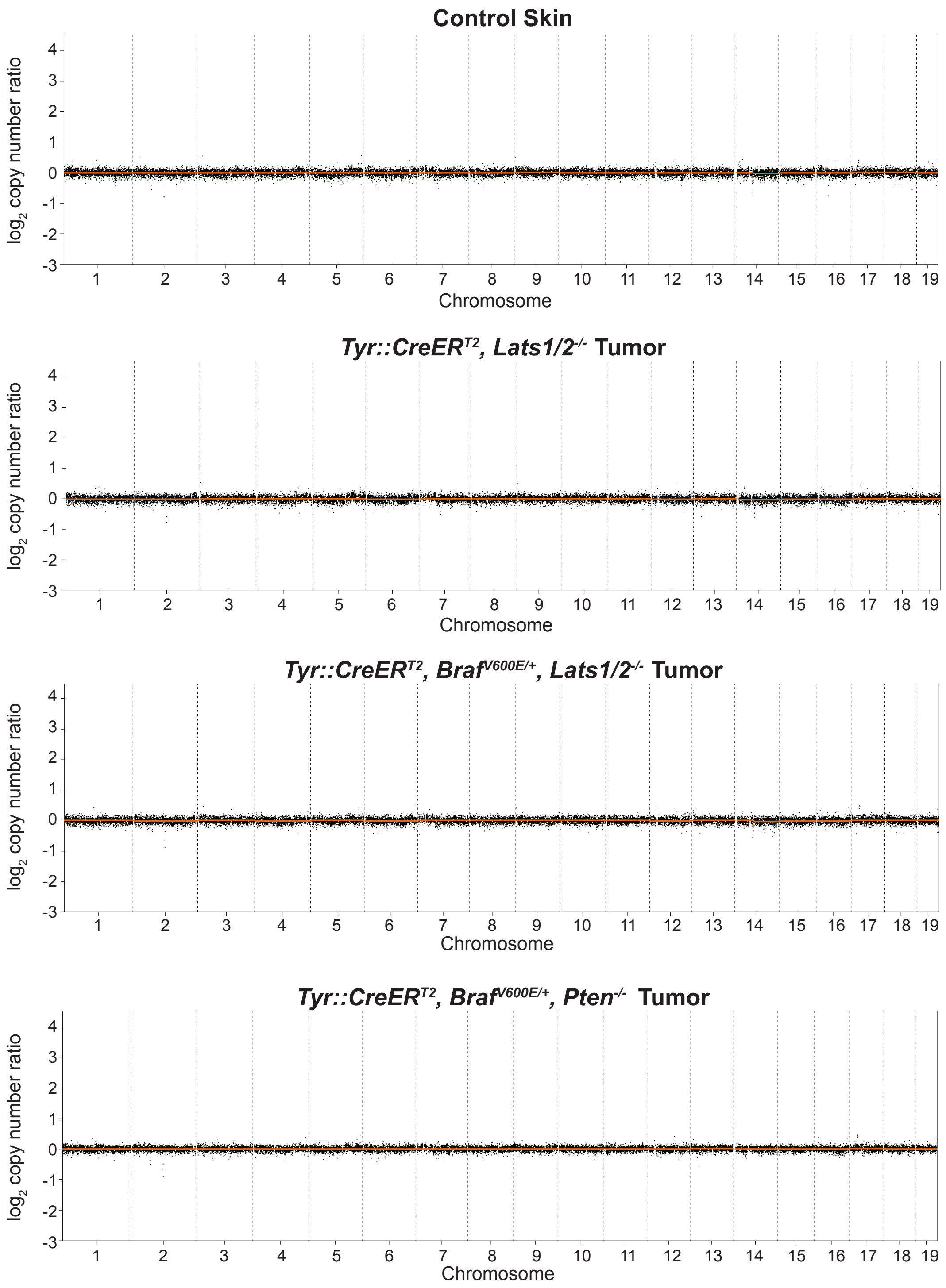

Control Skin
Tyr::CreER^{T2}, Lats1/2$^{-/-}$ Tumor
Tyr::CreER^{T2}, Braf$^{V600E/+}$, Lats1/2$^{-/-}$ Tumor
Tyr::CreER^{T2}, Braf$^{V600E/+}$, Pten$^{-/-}$ Tumor
log_2 copy number ratio
Chromosome
4
3
2
1
0
-1
-2
-3
1
2
3
4
5
6
7
8
9
10
11
12
13
14
15
16
17
18
19

Supplemental Figure 8: Murine Melanomas Lack Significant Aneuploidy (a) Representative copy number plots of ULP-WGS copy number analysis of control skin (*Tyr::CreER*$^{T2-/-}$) or tumors from indicated genotypes (control n = 1, *Lats1/2*$^{-/-}$ n = 3, *Braf*^{V600E}*/Lats1/2*$^{-/-}$ n = 2, *Braf*^{V600E}*/PTEN*$^{-/-}$ n = 3). Source Data are provided as a Source Data file.

Supplementary Table 1: YAP/TAZ Gene Set

Gene	Derived from:	Gene	Derived from:
AGFG2	Cordenonsi	IGFBP3	Wang et al. 2018
AMOTL2	Cordenonsi / Wang et al. 2018	ITGB2	Cordenonsi
ANKRD1	Cordenonsi / Wang et al. 2018	ITGB5	Cordenonsi
ARHGEF17	Wang et al. 2018	LATS2	Wang et al. 2018
ASAP1	Cordenonsi / Wang et al. 2018	LHFPL6	Cordenonsi
AXL	Cordenonsi / Wang et al. 2018	MARCKS	Cordenonsi
BICC1	Cordenonsi	MDFIC	Cordenonsi
BIRC5	Cordenonsi	MYOF	Wang et al. 2018
CAVIN2	Cordenonsi	NDRG1	Cordenonsi
CCDC80	Wang et al. 2018	NT5E	Wang et al. 2018
CDC20	Cordenonsi	NUAK2	Wang et al. 2018
CDKN2C	Cordenonsi	PDLIM2	Cordenonsi
CENPF	Cordenonsi	PHGDH	Cordenonsi
COL4A3	Cordenonsi	PMP22	Cordenonsi
CRIM1	Cordenonsi / Wang et al. 2018	PTPN14	Wang et al. 2018
CCN2 (CTGF)	Cordenonsi / Wang et al. 2018	RBMS3	Wang et al. 2018
CCN1 (CYR61)	Cordenonsi / Wang et al. 2018	SCHIP1	Cordenonsi
DAB2	Cordenonsi	SERPINE1	Cordenonsi
DDAH1	Cordenonsi	SGK1	Cordenonsi
DLC1	Cordenonsi	SH2D4A	Cordenonsi
DOCK5	Wang et al. 2018	SHCBP1	Cordenonsi
DUSP1	Cordenonsi	SLIT2	Cordenonsi
DUT	Cordenonsi	STMN1	Cordenonsi
ECT2	Cordenonsi	TGFB2	Cordenonsi / Wang et al. 2018
EMP2	Cordenonsi	TGM2	Cordenonsi
ETV5	Cordenonsi	THBS1	Cordenonsi
F3	Wang et al. 2018	TK1	Cordenonsi
FGF2	Cordenonsi	TNNT2	Cordenonsi
FJX1	Wang et al. 2018	TNS1	Cordenonsi
FLNA	Cordenonsi	TOP2A	Cordenonsi
FOXF2	Wang et al. 2018	TSPAN3	Cordenonsi
FSCN1	Cordenonsi		
FSTL1	Cordenonsi		
GADD45A	Wang et al. 2018		
GADD45B	Cordenonsi		
GAS6	Cordenonsi		
GGH	Cordenonsi		
GLS	Cordenonsi		
HEXB	Cordenonsi		
HMMR	Cordenonsi		

Supplementary Table 2: Hippo Component Gene Set

Genes
AMOT
AMOTL1
AMOTL2
CASP3
DVL2
LATS1
LATS2
MOB1A
MOB1B
NPHP4
SAV1
STK3
STK4
TJP1
TJP2
WWC1
WWTR1
YAP1
YWHAB
YWHAE
TEAD1
TEAD2
TEAD3
TEAD4
