# Supplementary material for: Inactivation of the Hippo tumor suppressor pathway promotes melanoma
Source: Nat Commun. 2022 Jun 29;13:3732. doi: 10.1038/s41467-022-31399-w (PMC9243107; doi:10.1038/s41467-022-31399-w)
Supplement: Supplementary file 3 — Reporting Summary [file 41467_2022_31399_MOESM3_ESM.pdf]

nature portfolio

Corresponding author(s): Neil Ganem

Last updated by author(s): May 5, 2022

Reporting Summary

Nature Portfolio wishes to improve the reproducibility of the work that we publish. This form provides structure for consistency and transparency in reporting. For further information on Nature Portfolio policies, see our Editorial Policies and the Editorial Policy Checklist.

Statistics

For all statistical analyses, confirm that the following items are present in the figure legend, table legend, main text, or Methods section.

n/a	Confirmed	
☐	☒	The exact sample size (*n*) for each experimental group/condition, given as a discrete number and unit of measurement
☐	☒	A statement on whether measurements were taken from distinct samples or whether the same sample was measured repeatedly
☐	☒	The statistical test(s) used AND whether they are one- or two-sided<br>*Only common tests should be described solely by name; describe more complex techniques in the Methods section.*
☐	☒	A description of all covariates tested
☐	☒	A description of any assumptions or corrections, such as tests of normality and adjustment for multiple comparisons
☐	☒	A full description of the statistical parameters including central tendency (e.g. means) or other basic estimates (e.g. regression coefficient) AND variation (e.g. standard deviation) or associated estimates of uncertainty (e.g. confidence intervals)
☐	☒	For null hypothesis testing, the test statistic (e.g. *F*, *t*, *r*) with confidence intervals, effect sizes, degrees of freedom and *P* value noted<br>*Give P values as exact values whenever suitable.*
☒	☐	For Bayesian analysis, information on the choice of priors and Markov chain Monte Carlo settings
☒	☐	For hierarchical and complex designs, identification of the appropriate level for tests and full reporting of outcomes
☒	☐	Estimates of effect sizes (e.g. Cohen's *d*, Pearson's *r*), indicating how they were calculated

Our web collection on statistics for biologists contains articles on many of the points above.

Software and code

Policy information about availability of computer code

Data collection	Image collection: NIS-Elements AR (Version 4.13.04) or Echo Revolve Microscope (Version 2.2.4, App Version 3.7.5 Build 6095). Luminescence Analysis Software: BMG Labtech Optima v2.20R2. Immunoblot imaging software: ImageLab v5.2.1 v11.
Data analysis	Data were analyzed in Prism 9 (Version 9.3.1), ImageJ (Version 1.51), NIS-Elements AR (Version 4.13.04), ImageLab (Version 6.1), GSEA (v4.1.0)

For manuscripts utilizing custom algorithms or software that are central to the research but not yet described in published literature, software must be made available to editors and reviewers. We strongly encourage code deposition in a community repository (e.g. GitHub). See the Nature Portfolio guidelines for submitting code & software for further information.

Data

Policy information about availability of data

All manuscripts must include a data availability statement. This statement should provide the following information, where applicable:

- Accession codes, unique identifiers, or web links for publicly available datasets
- A description of any restrictions on data availability
- For clinical datasets or third party data, please ensure that the statement adheres to our policy

TCGA (https://www.cancer.gov/tcga) and MSKCC data sets can be obtained from https://portal.gdc.cancer.gov. Gene set data used in the study are publicly available: GSE61750 https://www.ncbi.nlm.nih.gov/geo/query/acc.cgi?acc=gse61750, GSE154679 https://www.ncbi.nlm.nih.gov/geo/query/acc.cgi?acc=GSE154679 . Sequencing data generated in this study is publicly available on an NCBI database, the Sequence Read Archive (SRA), accession code PRJNA835203. The authors declare that all other data supporting this study are either available within the article, supplementary figures, or available from the authors upon request.

Field-specific reporting

Please select the one below that is the best fit for your research. If you are not sure, read the appropriate sections before making your selection.

☒ Life sciences ☐ Behavioural & social sciences ☐ Ecological, evolutionary & environmental sciences

For a reference copy of the document with all sections, see nature.com/documents/nr-reporting-summary-flat.pdf

Life sciences study design

All studies must disclose on these points even when the disclosure is negative.

Sample size	Sample sizes for cell based assays were chosen according to the standards of the field (generally at least three independent biological replicates for each condition) which gave sufficient statistical power for the effect sizes of interest. They were not predetermined based upon statistical methods given the inability to predict effect size for the cell experiments employed herein. Minimum sample size for animal studies were able to be calculated using Boston University's Research Support and Compliance sample size calculator with an alpha of 0.05 and power of 0.95.
Data exclusions	No data were excluded.
Replication	Where statistical comparisons were made, experiments were performed with at least three independent biological replicates to ensure reproducibility. All experiments were consistently reproducible. All graphs include individual data points from biological replicates. The calculated minimum sample size was used for all animal studies where statistical comparisons were made, with at least three mice per group.
Randomization	Experimental groups involving cell culture, analysis of previously collected patient data, and animal models were not randomized. Although allocation was not random, all experiments were performed with appropriate negative and positive controls in keeping with the standards of the field.
Blinding	Investigators were blinded for live cell and fixed cell imaging studies and analysis. Investigators were blinded to murine genotypes when assessing tumor growth. For all other experimental modalities blinding was not performed as the experimental manipulations required knowledge of cell lines, but all experiments were performed and analyzed using unbiased methodology. Experiments were reproduced by multiple investigators whenever possible.

Reporting for specific materials, systems and methods

We require information from authors about some types of materials, experimental systems and methods used in many studies. Here, indicate whether each material, system or method listed is relevant to your study. If you are not sure if a list item applies to your research, read the appropriate section before selecting a response.

Materials & experimental systems

n/a	Involved in the study	
☐	☒	Antibodies
☐	☒	Eukaryotic cell lines
☒	☐	Palaeontology and archaeology
☐	☒	Animals and other organisms
☒	☐	Human research participants
☒	☐	Clinical data
☒	☐	Dual use research of concern

Methods

n/a	Involved in the study	
☒	☐	ChIP-seq
☒	☐	Flow cytometry
☒	☐	MRI-based neuroimaging

Antibodies

Antibodies used	Immunofluorescence (cells):<br>Santa Cruz Biotechnology: YAP 63.7 (detects both YAP/TAZ, sc-101199)<br>Immunofluorescence (tissue):<br>Cell Signaling Technologies: YAP (1A12) #12395, YAP/TAZ (D24E4) #8418, YAP (D8H1X) #14074<br>Abcam: gp100 (ab137078), SOX10 (Rabbit, ab180862), SOX10 (Mouse, ab216020)<br>Immunohistochemistry:<br>Cell Signaling Technologies: YAP/TAZ (D24E4) #8418, YAP (D8H1X) #14074, GFP (D5.1, cross reacts with YFP) #2956, phospho-p44/42 ERK1/2 (Thr202/Tyr204) #9101<br>Abcam: gp100 (ab137078), SOX10 (Rabbit, ab180862), SOX10 (Mouse, ab216020), MelanA (ab210546)<br>Dako: S100 (IS504), pre-diluted by manufacturer<br>Immunoblotting:<br>Cell Signaling Technologies: B-Raf (D9T6S), phospho-p44/42 ERK1/2 (Thr202/Tyr204) #9101, p-44/42 MAPK (ERK1/2) #9102, RSK1/RSK2/RSK3 (3D27) #9355, phospho-p90RSK (Ser380) (D3H11) #11989, GAPDH (14C10) #2118, YAP (D8H1X) #14074, LATS1 (C66B5) #3477, phospho-LATS1 (Thr1079) (D57D3) #8654, TAZ (E8E9G) #83669, TAZ (V386) #4883, phospho-S6 (Ser235/236) (D57.2.2E) XP

	#4858, PTEN (138G6) #9559, p53 (1C12) #2524, phospho-Chk1 (Ser345) #2341<br>Abcam: Vinculin (ab18058)<br>Invitrogen: BRAFV600E (RM8 Clone) #MA5-24661<br>Bethyl Laboratories: LATS1/LATS2 (A300-479A)<br>Santa Cruz Biotechnology: Chk1 (G-4) sc-8408, S6 (C-8) sc-74459, N-Ras (F155) sc-31<br>Spring Biosciences: BRAFV600E (VE1 Clone) # E19290<br>Cytoskeleton: RhoA (ARH05)
Validation	All antibodies are commercially available and validated by their manufacturer.<br>Antibody validation statements from each commercial manufacturer is included below.<br><br>Cell Signaling Technologies:<br>Cell Signaling Technology (CST) provides the highest quality primary and secondary antibodies available for western blotting. CST™ antibodies are produced in-house and validated extensively according to a rigorous protocol. Antibody validation includes: specificity testing on cell and tissue extracts with documented protein expression levels - not just on recombinant protein; specificity confirmation through the use of siRNA transfection or knockout cell lines; specificity testing of antibodies directed against a post-translational modification by treatment of cell lines with growth factors, chemical activators, or inhibitors, which induce or inhibit target expression; phospho-specificity testing of phospho-antibodies by phosphatase treatment.<br><br>Abcam<br>Antibodies are validated in western blot using lysates from cells or tissues that we have identified to express the protein of interest. Once we have determined the right lysates to use, western blots are run and the band size is checked for the expected molecular weight. We will always run several controls in the same western blot experiment, including positive lysate and negative lysate. When possible, we also include knock-out (KO) cell lines as a true negative control for our western blots. We are always increasing the number of KO-validated antibodies we provide. In addition, we run old stock alongside our new stock. If we know the old stock works well, this also acts as a suitable positive control. If the western blot result gives a clear clean band and we are happy with the result from the control lanes, these antibodies will be passed and added to the catalog.<br><br>Dako<br>The staining performance of all FLEX RTU antibodies has been defined, tested and approved through collaboration with leading, international pathology experts.<br><br>Invitrogen<br>Invitrogen antibodies are currently undergoing a rigorous 2-part testing approach. Part one is target specificity verification which helps ensure the antibody will bind to the correct target. Our antibodies using at least one of the following methods to ensure proper functionality in researchers experiments: knockout, knockdown, independent antibody verification, cell treatment, relative expression, neutralization, peptide array, SNAP-ChIP, immunoprecipitation-mass spectrometry. Part 2 is functional application validation. These tests help ensure the antibody works in a particular application of interest, which may include; Western blotting, flow cytometry, ChIP, immunofluorescence imaging, immunohistochemistry.<br><br>Bethyl Laboratories<br>By analyzing western blots of immunoprecipitates, in conjunction with a western blot of the whole-cell lysate, we can verify the mobility of the target protein. Using multiple dilutions and a broad spectrum of whole-cell lysates, our scientists can verify selective binding as well as the antibody's specificity, reproducibility, and sensitivity. At the conclusion of this two-phase process, only antibodies exhibiting the following characteristics qualify to be released: Specific recognition of the target protein, Selective recognition of the target protein, Acceptable sensitivity, and Reproducibility.<br><br>Spring Biosciences<br>Now owned by Abcam, please see abcam's validation statement.<br><br>Cytoskeleton<br>Each antibody is developed in house and rigorously tested for in western blot, immunofluorescence, immunoprecipitation, and ELISA.

Eukaryotic cell lines

Policy information about cell lines

Cell line source(s)	Mel-ST cells were a gift from and originally derived in the lab of Dr. Robert Weinberg, HEK293A cells were sourced from Invitrogen, hTERT-BJ fibroblasts were obtained from ATCC and were originally deposited by JR Smith, D4M.3A mouse tumor cells were a gift of and originally derived by the lab of Dr. Constance Brinckerhoff
Authentication	All cell lines were either purchased directly from the above corporations or directly mailed from the gifting labs, but we did not independently authenticate them
Mycoplasma contamination	All cell lines were routinely tested for mycoplasma contamination utilizing ABM's mycoplasma PCR detection kit (G238). All cell lines used for experiments in this study were confirmed negative for mycoplasma contamination.
Commonly misidentified lines<br>(See ICLAC register)	None of the cell lines are commonly misidentified.

Animals and other organisms

Policy information about studies involving animals; ARRIVE guidelines recommended for reporting animal research

Laboratory animals	Murine Studies:<br>mus musculus, C57BL/6, female and male, various ages dependent upon experiment. Please see Methods section in article for further details. All other mouse strains were sourced from Jackson Laboratory and then further bred in-house according to protocols approved by Boston University IACUC: Tyr::CreERT2 (Jax # 012328), Lats1f/f (Jax # 024941), Lats2f/f (Jax # 025428), BRAFV600E (Jax # 017837), R26-YFPLSL (Jax # 006148).<br><br>Zebrafish studies:<br>Zebrafish mitfa(lf) mutant animals were used with various constructs as detailed in methods. Equal numbers of male and female fish were utilized and zebrafish were at an age of 4-5 days of development when scored for melanocyte rescue and then rescued animals were monitored for melanoma. Please see methods and cited prior studies for further details.
Wild animals	No wild animals were used in this study.
Field-collected samples	No field-collected samples were used in this study.
Ethics oversight	All murine experiments were conducted according to protocols approved by the Institutional Animal Care and Use Committee (IACUC) at Boston University (Protocol # PROTO201800236). All zebrafish experiments were conducted according to protocols approved by the Institutional Animal Care and Use Committee (IACUC) at University of Massachusetts Medical School (Protocol A-2171).

Note that full information on the approval of the study protocol must also be provided in the manuscript.
